# Supplementary material for: Hermetia illucens frass improves the physiological state of basil (Ocimum basilicum L.) and its nutritional value under drought
Source: PLoS One. 2023 Jan 17;18(1):e0280037. doi: 10.1371/journal.pone.0280037 (PMC9844844; doi:10.1371/journal.pone.0280037)
Supplement: Attachment — Submitted filename: Response to Reviewers[8665].docx [file pone.0280037.s003.docx]

Dear Dr. Umakanta Sarker,

Thank you for your Editorial service towards the peer-review of our manuscript. The Authors are very grateful to the Reviewer for pointing out deficiencies to make this a stronger submission. We added the necessary information. We hope that the revised manuscript now meets the requirements for publication in *PLoS One*.

Respectfully yours,

Marcin Nowicki

**#Rewiever 1**

**Comment:** The work by Radzikowska et al. advances our understating the biological role for the frass fertilizer for increasing basil physiology and its nutritional status in the presence of insufficient water. The report is well and condensed, as well as technically may be appropriate for PLOS ONE. However, before being able to recommend acceptance, I invite authors to address the following amendments.

**Response:** Thank you for your helpful comments and suggestions for improvement; these made our report stronger.

**Comment:** 1. I made some corrections in the manuscript, please follow those.

**Response:** Thank you for your work. We genuinely appreciate it.

**Comment:** 2. Extensive editing of English language

**Response:** We carefully scanned and edited the Manuscript and accompanying files throughout, to the best of our collective command of English. We noted the often-missing spaces, likely introduced during the conversion to .pdf by the Submission system. We could not address the generic character of that comment without any specific passages needing “extensive editing” in the Reviewer’s opinion.

**Comment:** 3. Cite some references how frass improve plant physiology and quality.

**Response:** It has been added to the MS (69-80).
“Use of Hermetia illucens frass (HI frass) as an additive to the growing media in the soilless production of basil, lettuce, and tomato increased the yield (drought mass) [12]. Also, the study of the compost produced as a result of the bioconversion of coffee husks by the larvae of the H. illucens in the cultivation of lettuce showed a positive effect of such fertilizers on plant height, number of leaves, leaf area, and chlorophyll content [13]. Recently published data indicate that the use of insect frass as a soil component in plant cultivation provides nitrogen and other nutrients, which consequently increases plant biomass and nutrient content [11,14]. Quality of the HI frass is high in terms of N, P, and K levels [15]. Use of HI frass from larvae increased the soil organic matter and the residual nutrients content, as well as the enzymatic activity of dehydrogenase and β-glucosidase [16].

**Comment:** 4. Cite some references how drought affects negatively on physiology and quality of Basil.

**Response:** It has been added to the MS (99-111).

“Basil is an annual plant that can be successfully grown in warm and sunny conditions [30]. The optimal growth temperature ranges from 298 to 303 K [31]. Basil, however, is quite sensitive to drought stress [32]. Drought stress triggers a number of various physiological responses that have negative effects on plant growth and development. Plants are adapted to the short-term effect of the stress factor, whereas under prolonged drought stress, the rate of water absorption from the leaves is increased, which consequently reduces their hydration. This can result in closure of the stomata and in reduced cell enlargement and growth [33]. In addition, drought also causes a reduction in the activity of the electron transport chain, which leads to the accumulation of reactive oxygen species (ROS) that are toxic at elevated levels [34]. ROS may damage nucleic acids, proteins, photosynthetic pigments, and membrane lipids [34,35], and that damage may impair plant development, yield, and even lead to plant death.

**Comment:** 5. Materials and Methods should be clearer

**Response:** The description of the methods is based on the literature data and manufacturers' recommendations. We agree with the Reviewer that it could be clearer as it is very detailed and extensive. But, with the reviewer's consent, we would like to leave that section as currently described. Our accurate descriptions of the analytical methods allow their independent reproduction.

**Comment:** What was the water holding capacity of the experimental soil at 80% Field capcity and what was the soil pH?

**Response:** Soil pH was 6.2; this information has been added to the text (124). The water capacity was of lesser import for us, because the only differing factor throughout the experiment was the fertilization regime. Please note, data for full parallel characteristics under control (non-stressed; regularly watered) and drought (stressed) conditions are presented; these were generated in the same soil throughout.

**Comment:** ℃

**Response:** It means Celsius degree. It has been calculated to K, according to SI units.

**Comment:** Where is figure?

**Response:** The figure is attached as a separate file. It was successfully produced into the .pdf.
